# Supplementary material for: Isometric representations in neural networks improve robustness
Source: Sci Rep. 2025 Oct 21;15:36761. doi: 10.1038/s41598-025-20619-0 (PMC12541071; doi:10.1038/s41598-025-20619-0)
Supplement: Supplementary file 1 — Supplementary Information. [file 41598_2025_20619_MOESM1_ESM.pdf]

1 Appendix

1.1 Derivation for Lipschitz bound on the isometric loss gradient.

We start be writing down the isometric loss term:

$$\mathcal{L}_{ISO} = \frac{\beta}{N^2}||G \odot D_{\mathcal{M}} - G \odot D_{\Phi}||_F^2. \tag{1}$$

When taking the gradient we can rewrite it in terms of the distance generating functions $d(x_i, x_j)$ and $d(\phi(x_i), \phi(x_j))$. We need only consider distances in the set $(i,j)_G = \{i,j|G_{i,j} \neq 0\}$. Furthermore for simplicity of notation we will abbreviate $\Phi(x_i)$ to ϕ_i and use l to indicate one of the basis components (eg. a pixel of an image). Lastly for the purpose of readability, we will omit writing out the element-wise multiplication operator for the indexing matrix G. This gives the following:

$$\beta\nabla_x\mathcal{L}_{ISO} = \frac{\beta}{N^2}\nabla_x \sum_{(i,j)_G}(d(x_i,x_j) - d(\phi_i,\phi_j))^2. \tag{2}$$

By expanding the squared brackets and applying the chain and product rules, the expression becomes:

$$\frac{2\beta}{N^2}\sum_{(i,j)_G}[d(x_i,x_j)\nabla_x d(x_i,x_j) - d(x_i,x_j)\nabla_x d(\phi_i,\phi_j) - \\ d(\phi_i,\phi_j)\nabla_x d(x_i,x_j) + d(\phi_i,\phi_j)\nabla_x d(\phi_i,\phi_j)], \tag{3}$$

rearranging these terms we get:

$$\frac{2\beta}{N^2}\sum_{(i,j)_G}\{d(x_i,x_j)[\nabla_x d(x_i,x_j) - \nabla_x d(\phi_i,\phi_j)] + d(\phi_i,\phi_j)[\nabla_x d(\phi_i,\phi_j) - \nabla_x d(x_i,x_j)]\} = \\ \frac{2\beta}{N^2}\sum_{(i,j)_G}\{[d(x_i,x_j) - d(\phi_i,\phi_j)][\nabla_x d(x_i,x_j) - \nabla_x d(\phi_i,\phi_j)]\}. \tag{4}$$

In order to continue it helps to compute the derivatives of the distance functions with respect to the basis $\{z_1, .., z_n\}$ in which the input data is described, this gives,

$$\nabla_{x_i} d(x_i,x_j) = \frac{x_i - x_j}{d(x_i,x_j)}, \\ \nabla_{x_i} d(\phi_i,\phi_j) = \sum_k \left[\frac{\phi_i - \phi_j}{d(\phi_i,\phi_j)}\right]_k \left[\frac{\partial \phi_i}{\partial z^l}\right]_k = \frac{\phi_i - \phi_j}{d(\phi_i,\phi_j)}\frac{\partial \phi_i}{\partial z^l}, \tag{5}$$

where after the summation, both gradients live in a tangent space in the input domain $\mathbb{R}^d$. After substituting these terms, we can write a new expression,

$$\frac{2\beta}{N^2}\sum_{(i,j)_G}\{[d(x_i,x_j) - d(\phi_i,\phi_j)]\left[\frac{x_i - x_j}{d(x_i,x_j)} - \frac{\phi_i - \phi_j}{d(\phi_i,\phi_j)}\frac{\partial \phi_i}{\partial x^l}\right]. \tag{6}$$

At this point it helps to compress our notation further. We redefine the difference between the two distance matrices as $H_{i,j} = d(x_i, x_j) - d(\phi_i, \phi_j)$, the difference between the mapping at two different points as $J_{i,j} = \frac{x_i - x_j}{d(x_i,x_j)}$ or $J^{\phi}_{i,j} = \frac{\phi_i - \phi_j}{d(\phi_i,\phi_j)}$. Given these new identifications we obtain the final form of the gradient as:

$$\beta \nabla_x \mathcal{L}_{ISO} = \frac{2\beta}{N^2} \sum_{(i,j)_G} [H_{i,j} J_{i,j} - H_{i,j} J^{\phi}_{i,j} \nabla_x \phi_i]. \tag{7}$$

For a local isometry, each component of $\nabla_x \phi_i$ is bounded by $K > 1$. With this in mind we arrive at the final inequality, showing that as long as an isometry is found during learning the isometric loss gradient is bounded:

$$\beta ||\nabla_x \mathcal{L}_{ISO}||_\infty \leq \frac{2\beta}{N^2} \sum_{(i,j)_G} [H_{i,j} J_{i,j} - H_{i,j} J^{\phi}_{i,j} K]. \tag{8}$$

□

1.2 Additional figures

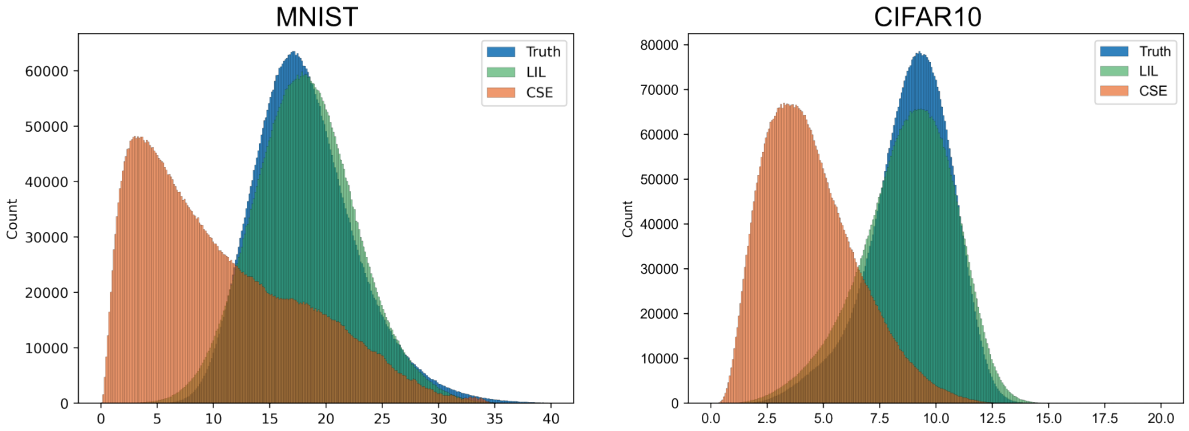

Figure 1: Histograms of the distances between the embeddings in the last hidden layer for all pairs of samples that share the same class label. Both LIL results use $\beta = 1$.
